# Deletion of IL-4Rα on CD4 T Cells Renders BALB/c Mice Resistant to Leishmania major Infection

**DOI:** 10.1371/journal.ppat.0030068

**Published:** 2007-05-11

**Authors:** Magdalena Radwanska, Antony J Cutler, J. Claire Hoving, Stefan Magez, Christoph Holscher, Andreas Bohms, Berenice Arendse, Richard Kirsch, Thomas Hunig, James Alexander, Paul Kaye, Frank Brombacher

**Affiliations:** 1 Division of Immunology, Institute of Infectious Disease and Molecular Medicine, University of Cape Town, Cape Town, South Africa; 2 VIB, Vrije Universiteit Brussel, Brussels, Belgium; 3 Institute for Virology and Immunobiology, University of Wurzburg, Wurzburg, Germany; 4 Strathclyde Institute of Pharmacy and Biomedical Sciences, University of Strathclyde, Glasgow, United Kingdom; 5 Immunology and Infection Unit, Department of Biology, University of York, York, United Kingdom; Center for Global Health and Diseases, United States of America

## Abstract

Effector responses induced by polarized CD4^+^ T helper 2 (Th2) cells drive nonhealing responses in BALB/c mice infected with *Leishmania major.* Th2 cytokines IL-4 and IL-13 are known susceptibility factors for L. major infection in BALB/c mice and induce their biological functions through a common receptor, the IL-4 receptor α chain (IL-4Rα). IL-4Rα–deficient BALB/c mice, however, remain susceptible to L. major infection, indicating that IL-4/IL-13 may induce protective responses. Therefore, the roles of polarized Th2 CD4^+^ T cells and IL-4/IL-13 responsiveness of non-CD4^+^ T cells in inducing nonhealer or healer responses have yet to be elucidated. CD4^+^ T cell–specific IL-4Rα (Lck^cre^IL-4Rα^−/lox^) deficient BALB/c mice were generated and characterized to elucidate the importance of IL-4Rα signaling during cutaneous leishmaniasis in the absence of IL-4–responsive CD4^+^ T cells. Efficient deletion was confirmed by loss of IL-4Rα expression on CD4^+^ T cells and impaired IL-4–induced CD4^+^ T cell proliferation and Th2 differentiation. CD8^+^, γδ^+^, and NK–T cells expressed residual IL-4Rα, and representative non–T cell populations maintained IL-4/IL-13 responsiveness. In contrast to IL-4Rα^−/lox^ BALB/c mice, which developed ulcerating lesions following infection with *L. major,* Lck^cre^IL-4Rα^−/lox^ mice were resistant and showed protection to rechallenge, similar to healer C57BL/6 mice. Resistance to L. major in Lck^cre^IL-4Rα^−/lox^ mice correlated with reduced numbers of IL-10–secreting cells and early IL-12p35 mRNA induction, leading to increased delayed type hypersensitivity responses, interferon-γ production, and elevated ratios of inducible nitric oxide synthase mRNA/parasite, similar to C57BL/6 mice. These data demonstrate that abrogation of IL-4 signaling in CD4^+^ T cells is required to transform nonhealer BALB/c mice to a healer phenotype. Furthermore, a beneficial role for IL-4Rα signaling in L. major infection is revealed in which IL-4/IL-13–responsive non-CD4^+^ T cells induce protective responses.

## Author Summary

Leishmaniasis is a disease induced by a protozoan parasite and transmitted by the sandfly. Several forms of infection are identified, and the different diseases have wide-ranging symptoms from localized cutaneous sores to visceral disease affecting many internal organs. Animal models of human cutaneous leishmaniasis have been established in which disease is induced by infecting mice subcutaneously with *Leishmania major.* Different strains of inbred mice have been found to be susceptible or resistant to L. major infection. “Healer” C57BL/6 mice control infection with transient lesion development. The protective response to infection in this strain is dominated by type 1 cytokines inducing parasite killing by nitric oxide. Conversely, “nonhealer” BALB/c mice are unable to control infection and develop nonhealing lesions associated with a dominant type 2 immune response driven by cytokines IL-4 and IL-13. However, mice deficient in IL-4/IL-13 signaling are not protected against development of cutaneous leishmaniasis. Here we describe a BALB/c mouse where the ability to polarize to a dominant type 2 response is removed by cell-specific deletion of the receptor for IL-4/IL-13 on CD4^+^ T cells. These mice are resistant to L. major infection similar to C57BL/6 mice, which highlights the role of T helper 2 cells in driving susceptibility and the protective role of IL-4/IL-13 signaling in non-CD4^+^ T cells in BALB/c mice.

## Introduction

Experimental Leishmania major infection is widely used to explore the control of T helper 1 (Th1)/Th2 differentiation and elucidate mechanisms underlying susceptibility/resistance to intracellular microbial infection [1,2]. Typically, susceptible BALB/c mice infected subcutaneously with L. major develop severe pathology, manifested by progressive lesion development, necrosis, and death, while resistant C57BL/6 mice are able to control and heal dermal lesions [3]. Nonhealing disease in BALB/c mice is associated with a Th2 response characterized by secretion of mainly IL-4, IL-5, IL-9, and IL-13 [2,4–7], high anti-*Leishmania* antibody titres, arginase-1 production by macrophages [8,9] and visceral dissemination of parasites [10]. In contrast, resistance to L. major infection is mediated by development of a protective Th1 response, in which sustained IL-12 production, interferon-γ (IFN-γ) release and macrophage killing via effector nitric oxide (NO) production catalyzed by inducible NO synthase (iNOS) underlie protective responses [9,11–14].

CD4 T cell–derived cytokines drive L. major responses, and, as such, events that control T cell differentiation in response to L. major appear to be critical for disease outcome [15]. Disruption of Th1 differentiation by neutralization of IL-12 renders resistant C57BL/6 mice susceptible, whereas susceptible BALB/c mice treated with IL-12 become resistant to L. major infection [12]. IL-12 production must be sustained to control infection [13]. While both resistant and susceptible mice produce IL-4 early after infection [16,17], production of this cytokine is sustained in susceptible mice and transient in resistant mice [16–18]. Neutralization of IL-4 allowed control of L. major infection in BALB/c mice [19]. Subsequent studies in knockout mice proved that IL-4 was indeed important but not the sole mediator of susceptibility in BALB/c mice. L. major infection was controlled in BALB/c IL-4^−/−^ mice, but parasite burdens remained greater than those of resistant animals [6,20]. These observations remain controversial, with some laboratory strains developing IL-4–independent susceptibility and indicating that further factors are involved [21]. IL-13 has been implicated as a susceptibility factor in L. major infection [4]. Susceptible IL-13 transgenic C57BL/6 mice develop impaired IL-12 and IFN-γ production during acute leishmaniasis, while IL-13^−/−^ BALB/c mice remain comparatively resistant [4,22]. IL-13 can influence Th1 differentiation by modulating macrophage function and suppressing secretion of NO, IL-12, and/or IL-18 [22,23], partially attributed to IL-4/IL-13 activated alternative macrophages (aaMphs), recently demonstrated in mice deficient for this activation status [9,24].

IL-4 and IL-13 share a common signaling pathway through the IL-4 receptor α (IL-4Rα) chain. A functional IL-4R (type I) requires assembly of IL-4Rα with a γc chain, while interaction of IL-4Rα with an IL-13Rα1 subunit leads to formation of a functional IL-13 receptor (type II) [25]. IL-4Rα–deficient mice therefore lack responsiveness to IL-4 and IL-13. Careful analysis of footpad swelling and lesion development showed that initial control of L. major infection is equivalent in IL-4^−/−^ and IL-4Rα^−/−^ BALB/c mice. However, in contrast to IL-4^−/−^ mice, IL-4Rα^−/−^ mice develop progressive chronic disease. These data clearly indicate a protective role for IL-13 signaling in protection against chronic L. major infection, at least in the absence of IL-4 responsiveness [20].

Expression of IL-4Rα reflects the pleiotropic nature of IL-4 biology, as this receptor subunit is expressed upon a wide range of cells [26]. Given the central role of T cells in controlling L. major infection [15] and of IL-4 in driving Th2 responses [27], CD4^+^ T cell–specific IL-4Rα knockout mice were generated to elucidate the role of IL-4Rα–mediated signaling in CD4^+^ T cells independently of non-CD4^+^ T cell populations. Our results demonstrate a successful generation of transgene-bearing hemizygous Lck^cre^IL-4Rα^−/lox^ BALB/c mice that have effective deletion of IL-4Rα on CD4^+^ T cells, an incomplete deletion on CD8^+^ T cells and other T cell subpopulations, and normal expression on non–T cells. Lck^cre^IL-4Rα^−/lox^ mice infected with L. major developed a healing disease phenotype and clinical immunity similar to genetically resistant C57BL/6 mice. Consequently, our studies demonstrate that impairment of IL-4Rα–dependent Th2 polarized CD4^+^ T cells in the presence of IL-4/IL-13–responsive non-CD4^+^ T cells is required to transform nonhealer BALB/c mice to a healer phenotype.

## Results

### Genotypic and Phenotypic Characterization of Lck^cre^IL-4Rα^−/lox^ BALB/c Mice

**Figure 1:**
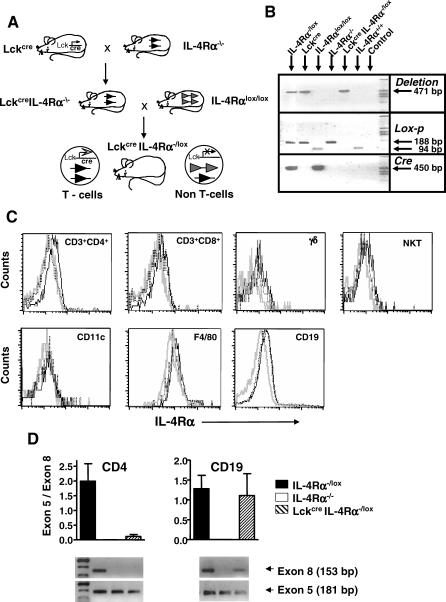
Generation of Lck^cre^IL-4Rα^−/lox^ Mice (A) Mouse breeding strategy. IL-4Rα^lox/lox^ BALB/c mice were intercrossed with transgenic BALB/c mice expressing Cre-recombinase under control of the Lck promoter and IL-4Rα^−/−^ BALB/c mice to generate Lck^cre^IL-4Rα^−/lox^ mice. The “loxed” IL-4Rα allele, gray arrows; deleted allele, black arrows. (B) Genotyping of Lck^cre^IL-4Rα^−/lox^ mice. The deleted IL-4Rα PCR yields a product of 471 bp, LoxP, 188 bp (loxed), and 94 bp (WT), and Cre-specific a 450-bp product. (C) Phenotypic analysis. WT (solid line), IL-4Rα^−/−^ (gray line), and Lck^cre^IL-4Rα^−/lox^ BALB/c mice (dashed line) LN cells were stained for expression of IL-4Rα. T cell subsets were identified using anti-CD3, anti-CD4/CD8, or δ-TCR. B cells, anti-CD19. DCs, CD11^c^/I-A^d^. Macrophages, F4/80/I-A^d^. (D) Efficiency of IL-4Rα deletion. The ratio of IL-4Rα exon 5 and exon 8 alleles was determined by real-time PCR from genomic DNA purified from CD4^+^ or CD19^+^ cells. PCR products of amplified genomic DNA from real-time PCR reactions (75 cycles) were visualized on agarose gel. Data is representative of 2 independent experiments with triplicate values ± SD.

Recently established IL-4Rα^lox/lox^ BALB/c mice [24] were intercrossed with BALB/c mice expressing Cre-recombinase under control of the T cell–specific promoter Lck [28] and IL-4Rα^−/−^ BALB/c mice [20] to generate Lck^cre^IL-4Rα^−/lox^ mice (Figure 1A). IL-4Rα hemizygosity (^−/lox^) increases probability of Cre-mediated deletion of the “floxed” allele [24]. Lck^cre^IL-4Rα^−/lox^ mice were identified by PCR genotyping (Figure 1B). Fluorescence-activated cell sorter (FACS) analysis of IL-4Rα surface expression confirmed efficient deletion on CD3^+^CD4^+^ T cells from Lck^cre^IL-4Rα^−/lox^ mice when compared with IL-4Rα^−/−^ and IL-4Rα^−/lox^ BALB/c (WT) controls (geometric mean channel florescence [geo. mean]: WT = 18.11, IL-4Rα^−/−^ = 8.5, Lck^cre^IL-4Rα^−/lox^ = 9.48), but incomplete and variable deletion efficiency was observed on CD8^+^ T cells (Figure 1C and Figure S1) (geo. mean: WT = 18.69, IL-4Rα^−/−^ = 9.06, Lck^cre^IL-4Rα^−/lox^ = 13.96) and γδ^+^ (geo. mean: WT = 7.6, IL-4Rα^−/−^ = 3.15, Lck^cre^IL-4Rα^−/lox^ = 6.72) and NK–T cells (geo. mean: WT = 9.03, IL-4Rα^−/−^ = 5.25, Lck^cre^IL-4Rα^−/lox^ = 7.28; Figure 1C). The cellular specificity of IL-4Rα deletion was confirmed because B cells (CD19^+^), macrophages, and dendritic cells (DCs; Figure 1C) of Lck^cre^IL-4Rα^−/lox^ mice maintained expression of IL-4Rα. Efficiency of deletion of IL-4Rα in CD4^+^ T cells was analyzed at the genomic level by quantitative real-time PCR. The number of exon 5 alleles (both present in all cells) relative to exon 8 alleles (deleted in ^−/−^, one copy in ^−/lox^ mice) of IL-4Rα was determined in CD4^+^ T cells sorted to high purity. As expected, exon 8 was efficiently deleted in CD4^+^ T cells and B cells from IL-4Rα^−/−^ mice (Figure 1D). Confirming FACS analysis, efficient deletion of lox-p–flanked IL-4Rα exon 8 was observed in CD4^+^ T cells from Lck^cre^IL-4Rα^−/lox^ mice. Analysis revealed 0.114 copies of exon 8 were retained relative to exon 5, equating to 95.48% ± 5.8% deletion efficiency of exon 8 within the CD4^+^ T cell population. In agreement, no CD4^+^ T cell exon 8 product was visible following 75 PCR cycles (Figure 1D). An equivalent ratio of exon 8 versus exon 5 was maintained in CD19^+^ B cells in Lck^cre^IL-4Rα^−/lox^ mice compared with WT controls. These data provide evidence of efficient deletion of IL-4Rα in CD4^+^ T cells from Lck^cre^IL-4Rα^−/lox^ BALB/c mice.

### CD4^+^ T Cell–Specific Abrogation of IL-4Rα Function

**Figure 2:**
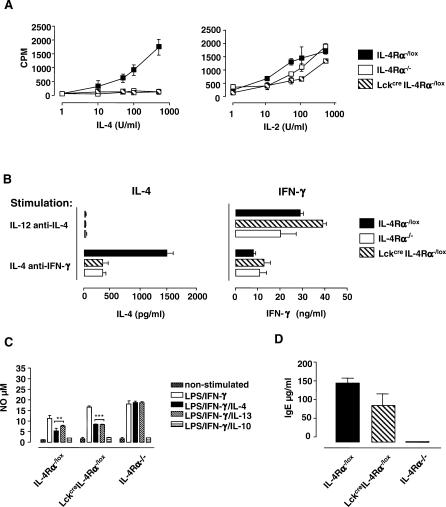
Functional Analysis of Lck^cre^IL-4Rα^−/lox^ Mice (A) Impaired proliferation in response to IL-4. [^3^H] thymidine incorporation by CD4^+^ T cells stimulated by serial dilutions of rIL-4 (left panel) or IL-2 (right panel). One of three representative experiments is shown with means of triplicate values ± SD. (B) Impaired Th2 differentiation of CD4^+^ T cells. CD4^+^ T cells were cultured in Th1 or Th2 polarizing conditions and IL-4 or IFN-γ secretion was measured by ELISA. A representative of one of three experiments is shown with means of triplicate values ± SD. (C) IL-4 and IL-13 suppress macrophage NO secretion in Lck^cre^IL-4Rα^−/lox^ mice. Peritoneal exudate cells were incubated with IL-4, IL-13, or IL-10 in combination with LPS/IFN-γ, LPS/IFN-γ alone, or medium alone. Nitrite levels were measured by Griess reaction. One of three experiments is shown with means of triplicate values ± SD. (**p < 0.01 or ***p < 0.001, LPS/IFN-γ versus LPS/IFN-γ + IL-4 or IL-13) (D) IgE production. Total IgE was measured in sera taken at 3 wk after infection and boosted with OVA (three mice per group). One representative experiment of two is shown.

IL-4 promotes proliferation of naive CD4^+^ T cells in vitro [29]. In order to assess functional impairment of IL-4Rα on CD4^+^ T cells from Lck^cre^IL-4Rα^−/lox^ mice, naive CD4^+^ T cells were stimulated with IL-4, and proliferation was measured by [^3^H] thymidine incorporation (Figure 2A). CD4^+^ T cells isolated from naive Lck^cre^IL-4Rα^−/lox^ BALB/c mice were unable to proliferate in response to IL-4, as were those from IL-4Rα^−/−^ mice. In contrast, WT CD4^+^ T cells showed dose-responsive proliferative responses to IL-4. Impairment of IL-4 signaling was IL-4Rα specific, as proliferative responses to IL-2, which shares a γc-chain with the type I IL-4R, were unaffected in all three strains (Figure 2A). Impairment of CD4^+^ T cell IL-4 responsiveness was further verified using the Th cell differentiation assay. Th1 versus Th2 differentiation of noncommitted CD4^+^ T cells may be achieved in vitro by treatment with either IL-12/anti–IL-4 or IL-4/anti–IFN-γ, respectively [29]. Naive CD4^+^ T cells stimulated with anti-CD3/CD28 and polarized with cytokine/neutralizing mAb treatment demonstrate that Th2 polarization, indicated by IL-4 production, was impaired in Lck^Cre^IL-4Rα^−/lox^ and IL-4Rα^−/−^ but not WT mice (Figure 2B). As expected, Th1 polarization was achieved in all three strains.

Functional macrophage IL-4Rα data from Lck^cre^IL-4Rα^−/lox^ mice were demonstrated in Figure 2C. NO production was suppressed by IL-4 and IL-13 in macrophages from Lck^cre^IL-4Rα^−/lox^ and WT mice (Figure 2C), but not IL-4Rα^−/−^ macrophages, showing IL-4Rα specificity. As a positive control, IL-10 suppressed NO production in all three strains. Production of IgE antibodies is strictly dependent on IL-4 signaling [30]. IL-4Rα responsiveness of B cells in Lck^cre^IL-4Rα^−/lox^ mice was demonstrated in Figure 2D. Antigen-induced IgE antibody was present at slightly reduced levels in OVA-immunized Lck^cre^IL-4Rα^−/lox^ mice when compared with those of WT mice, while IgE production was completely abrogated in IL-4Rα^−/−^ mice (Figure 2D). Together, these data provide evidence for effective impairment of IL-4Rα–mediated functions in Lck^cre^IL-4Ra^−/lox^ CD4^+^ T cells, but not in other lymphocyte subpopulations such as B cells and macrophages.

### Resistance to Acute and Chronic Leishmaniasis in Lck^cre^IL-4Rα^−/lox^ BALB/c Mice

**Figure 3:**
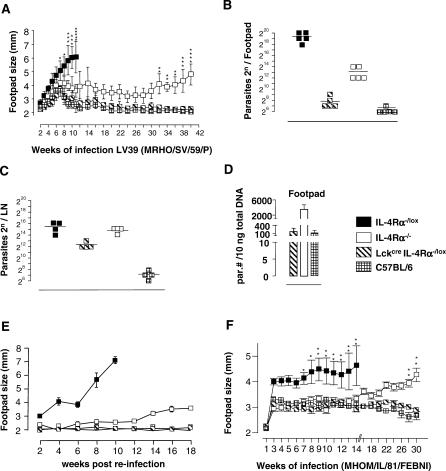
Lck^cre^IL-4Rα^−/lox^ Mice Control Footpad Swelling and Parasite Burden during Acute and Chronic L. major Infection (A) Lesion development. Footpad swelling was measured at weekly intervals in mice (five per group) infected with 2 × 10^6^ stationary phase L. major LV39 (MRHO/SV/59/P) metacyclic promastigotes into the hind footpad. Asterisk indicates ulceration or necrosis/mouse. A representative of one of five experiments is shown with mean values ± SD. (B) Week six footpad parasite load. Parasite load was determined by limiting dilution of single-cell suspensions from homogenized footpads at 6 wk after infection. (C) Week six LN parasite load. Parasite load was determined by limiting dilution of single-cell suspensions from the draining LNs at 6 wk after infection. (D) L. major parasite detection using real-time PCR at 36 wk after infection. Kinetoplast DNA was quantified from footpads at week 36 after infection. One of two representative experiments is shown, with values representing mean parasite number ± SD. (E) Lck^cre^IL-4Rα^−/lox^ BALB/c mice are resistant to reinfection. At 6 wk after infection with *L. major,* mice were reinfected in the contralateral hind footpad, and footpad swelling was monitored for 18 wk. Data are representative of two independent experiments. (F) Lck^cre^IL-4Rα^−/lox^ BALB/c mice are resistant to L. major (MHOM/IL/81/FEBNI). Lesion development: mice (four per group) were infected with 2 × 10^6^ stationary phase L. major (MHOM/IL/81/FEBNI) metacyclic promastigotes into the hind footpad. Asterisk indicates ulceration or necrosis per mouse. Footpad swelling was measured at weekly intervals up to week 14 and every 2 wk thereafter. A representative of one of two experiments is shown with mean values ± SD.

Controversy remains as to whether IL-4 [6,20,21] and/or IL-4Rα signaling [20,31] are key components of susceptibility to L. major infection. Polarized Th2 cells certainly play a significant role in contributing to susceptibility [32]. To investigate the consequence of CD4^+^ T cell–specific IL-4Rα unresponsiveness in leishmaniasis, mice were infected subcutaneously with 2 × 10^6^ stationary phase metacyclic promastigotes of L. major LV39 (MRHO/SV/59/P; Figure 3A). As expected, WT mice developed rapidly growing nonhealing lesions (Figure 3A) within 12 wks of infection and were unable to control parasite burden with high parasite numbers in the footpads (Figure 3B) and LNs (Figure 3C). IL-4Rα^−/−^ mice initially controlled infection with intermediate parasite load in the draining lymph nodes (LNs) and footpad. However, as previously described [20], global IL-4Rα deficiency does not confer resistance to L. major infection, as the mice progressed to develop necrotic lesions in the chronic phase (Figure 3A). In contrast, Lck^cre^IL-4Rα^−/lox^ mice were able to resolve infection with lesion growth comparable with resistant C57BL/6 mice (Figure 3A). Lck^cre^IL-4Rα^−/lox^ mice carried low parasite burdens in the footpad, with approximately 2,000-fold less parasites in the footpad compared with that of WT 6 wk after infection (Figure 3B), and maintained an intermediate parasite burden in the draining LNs when compared with C57BL/6 and WT mice (Figure 3C). Resistance to L. major infection in CD4^+^ T cell–specific IL-4Rα–deficient mice was profound, as parasite load in the footpad was equivalent to that observed in C57BL/6 mice at 36 wk after infection using PCR to detect kinetoplast DNA at the lesion site (Figure 3D). Lck^cre^IL-4Rα^−/lox^ mice were also shown to be resistant to reinfection. At 6 wk after L. major infection, mice were reinfected in the contralateral footpad. Lck^cre^IL-4Rα^−/lox^ mice were again comparable with genetically resistant C57BL/6 mice in lesion development, while L. major reinfection in WT mice progressed to necrosis in acute phase (Figure 3E). Lck^cre^IL-4Rα^−/lox^ mice were also resistant to the more virulent L. major (MHOM/IL/81/FEBNI) strain (Figure 3F), again with lesion kinetics comparable with that of C57BL/6 mice.

### Susceptibility to L. major Is Associated with IL-10 Production

**Figure 4:**
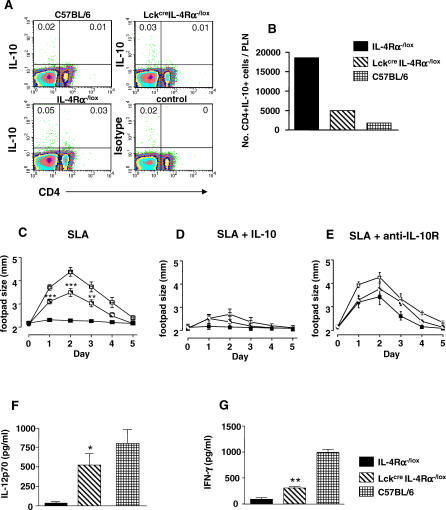
IL-10 Inhibits Protective Responses (A and B) Increased numbers of IL-10–secreting cells in IL-4Rα^−/lox^ mice. (A) CD4^+^ IL-10–secreting cells were identified by intracellular FACS in LN cells restimulated with SLA for 24 h in vitro from L. major–infected mice 6 wk after infection. Data represent one of two independent experiments (pool of eight popliteal LNs/group). (B) Total numbers of CD4^+^ IL-10–secreting cells per draining LN. (C–E) Susceptible mice exhibit poor DTH responses controlled by IL-10. At 6–8 wk after infection with *L. major,* mice (five mice per group) were injected in the contralateral hind footpad with (C) 10 μg SLA subcutaneously, (D) 10 μg SLA and 0.5 μg IL-10 subcutaneously, and (E) 10 μg SLA and 1.5 μg anti–IL-10R subcutaneously. Footpad swelling was monitored every 24 h for 5 d. (***p < 0.001, **p < 0.01 Lck^cre^IL-4Rα^−/lox^ versus WT). The data represent one of two independent experiments. (F) Increased IL-12p70 in DTH footpads of resistant mice. Lysates of footpads (four per group) taken 24 h after induction of DTH responses were analyzed for IL-12p70. The data represent the pool of two independent experiments (*p < 0.05, Lck^cre^IL-4Rα^−/lox^ versus WT). (G) Increased IFN-γ in DTH footpads of resistant mice. Lysates of footpads (four per group) taken 24 h after induction of DTH responses were analyzed for IFN-γ. The data represent the pool of two independent experiments (**p < 0.01, Lck^cre^IL-4Rα^−/lox^ versus WT).

IL-10 is a highly immunosuppressive cytokine, profoundly reducing NO production by macrophages (Figure 2C) [33], and is a susceptibility factor in L. major infection [31]. Intracellular cytokine staining revealed increased numbers of antigen-specific CD4^+^ IL-10–secreting T cells in the draining LNs of WT mice compared with C57BL/6 and Lck^cre^IL-4Rα^−/lox^ mice (Figure 4A and 4B). In order to examine an in vivo correlate demonstrating IL-10 inhibition of protective parasite specific responses, IL-12/IFN-γ–driven delayed type hypersensitivity (DTH) responses were investigated in L. major–infected mice. C57BL/6 develop sustained footpad swelling when challenged with soluble L. major antigen (SLA; Figure 4C), and Lck^cre^IL-4Rα^−/lox^ mice showed intermediate sustained swelling, whereas minimal DTH responses were observed in WT mice (Figure 4C). As expected, addition of IL-10 to SLA diminished DTH responses in all mice (Figure 4D). Neutralization of IL-10 function by blockade of IL-10R lifted suppression of the DTH in the low-responder WT mice on a par with DTH responses observed in the resistant strains (Figure 4E). Confirming that increased DTH responses observed in Lck^cre^IL-4Rα^−/lox^ mice resulted from increased Th1 responses, significant levels of IL-12p70 (Figure 4F) and IFN-γ (Figure 4G) were detected in footpad lysates taken from resistant mice, while little or no IL-12p70 or IFN-γ were induced in susceptible WT mice (Figure 4F and 4G).

### Increased Type 1 Responses in Lck^cre^IL-4Rα^−/lox^ BALB/c Mice

**Figure 5:**
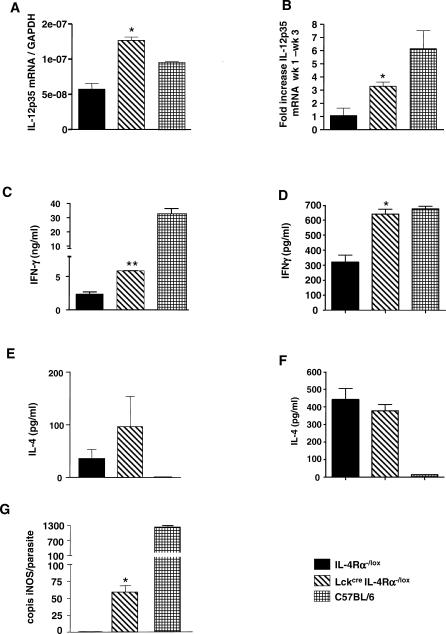
Type 1 Immunity Is Enhanced in Lck^cre^IL-4Rα^−/lox^ Mice in Response to L. major Infection (A and B) IL-12p35 mRNA expression is increased in resistant mice. IL-12p35 expression was determined by real-time RT-PCR from RNA prepared from pooled popliteal LN cells from week 3 L. major–infected mice (eight mice per group). Data are expressed as IL-12p35 copy numbers relative to *GAPDH* (A) or as fold increase in IL-12p35 mRNA from 3 wk versus 1 wk after infection (B). Mean ± SEM of three runs on the same sample. Data are representative of two independent experiments (*p < 0.05, Lck^cre^IL-4Rα^−/lox^ versus WT). (C and D) Increased IFN-γ production in resistant mice. (C) IFN-γ secretion by CD4 T cells cultured with fixed APCs and SLA. (**p < 0.01, Lck^cre^IL-4Rα^−/lox^ versus WT) and in (D) footpad homogenates (*p < 0.05, Lck^cre^IL-4Rα^−/lox^ versus WT) (homogenate data represent the pool of two independent experiments) from week 10 L. major–infected mice. (E and F) Maintained IL-4 production in resistant mice. (E) IL-4 secretion by CD4 T cells cultured with fixed APCs and SLA and in (F) footpad homogenates from week-ten L. major–infected mice. (G) iNOS production. iNOS mRNA copy number was calculated from footpad mRNA 6 wk after infection with *L. major.* At the same time, parasite DNA copy number was quantitated by PCR detecting L. major kinetoplast DNA. (*p < 0.05 Lck^cre^IL-4Rα^−/lox^ versus WT). The data represent the means of two individual experiments ± SEM.

IL-12 is a key protective cytokine involved in inducing protective responses following L. major infection [34]. We therefore examined IL-12 expression in Lck^cre^IL-4Rα^−/lox^ mice. Although IL-12p35 mRNA production was equivalent at 1 wk after infection (unpublished data), levels of IL-12p35 mRNA were increased in draining LNs of Lck^cre^IL-4Rα^−/lox^ and C57BL/6 mice at 3 wk after infection when compared with those of WT mice (Figure 5A). Levels of IL-12p35 mRNA increased from 1 wk to 3 wk after infection in resistant mice while remaining low in susceptible mice (Figure 5B). IFN-γ–driven iNOS production by macrophages is a key control mechanism in L. major infection [35]. CD4 T cell antigen–specific IFN-γ cytokine production was therefore examined. CD4 T cells from Lck^cre^IL-4Rα^−/lox^ mice induced 2.5-, 1.6-, and 2-fold more IFN-γ when compared with those from IL-4Rα^−/−^ and WT or IL-4Rα^−/lox^ mice at 10, 6, and 12 wk after infection (Figure 5C), respectively. Furthermore, greater IFN-γ levels were detected in footpad homogenates from infected Lck^cre^IL-4Rα^−/lox^ compared with WT mice at 10 wk after infection (Figure 5D). Importantly, IL-4Rα–independent IL-4 production was observed in Lck^cre^IL-4Rα^−/lox^ mice with similar levels of IL-4 production being observed in WT and Lck^cre^IL-4Rα^−/lox^ mice in antigen-specific CD4^+^ T cell restimulation (Figure 5E) and footpad lysates (Figure 5F). Consistently increased IFN-γ production had an influence on downstream macrophage effector functions. This was shown at 6 wk after infection, when more copies of iNOS mRNA/parasite were observed in resistant strains of mice (Figure 5G). Together, these data demonstrate that resistance to acute leishmaniasis in Lck^cre^IL-4Rα^−/lox^ mice is associated with an early induction of increased protective type 1 immunity and reduced suppression of responses by IL-10–secreting CD4^+^ T cells.

## Discussion

IL-4 and IL-13 share a common signaling pathway through the IL-4Rα chain [26], and as such the combined role of both cytokines can be studied in vivo in IL-4Rα^−/−^ mice. While IL-4 mediates multiple effects on T cells, murine T and B cells do not respond to IL-13 [7]. Generation of CD4^+^ T cell–specific IL-4Rα–deficient (Lck^cre^IL-4Rα^−/lox^) mice therefore allows investigation into the role of IL-4 signaling specifically on CD4^+^ T cells while maintaining IL-4/IL-13–mediated functions on non-CD4^+^ T cells. CD4^+^ T cell–specific IL-4Rα–deficient BALB/c mice were generated using the Cre/LoxP recombination system in BALB/c embryonic stem cells. Previous studies have shown efficiency of cell-specific Cre-mediated gene disruption may vary between 38%–85% depending on recombinase efficiency and promoter activity [36]. Efficiency of CD4^+^ T cell–specific IL-4Rα disruption (95.48%) was increased by using hemizygous WT mice instead of IL-4Rα^lox/lox^ as mating partners for transgenic Lck^Cre^ mice, thereby reducing the LoxP substrate for Cre-recombinase by 50%. FACS analysis showed efficient disruption of IL-4Rα gene expression in CD4^+^ T cells and incomplete deletion in CD8^+^ and NK–T cells with variable deletion efficiency. γδ T cells and non–T cells retained unaltered receptor expression in Lck^cre^IL-4Rα^−/lox^ mice. The data suggest that while the Lck promoter is functional and mediates deletion of loxP-flanked DNA sequences in CD4^+^, CD8^+^, and NK–T cell subsets, deletion is more efficient in CD4^+^ T cells using this promoter construct. Functional analysis further demonstrated effective and specific impairment of the IL-4 responsiveness of CD4 T cells, while B cells and macrophages retained IL-4– and IL-13–mediated functions. Thus, Lck^cre^IL-4Rα^−/lox^ mice are CD4^+^ T cell–specific IL-4Rα knockout mice, whereas all other cell types remain responsive to IL-4/IL-13.

Lck^cre^IL-4Rα^−/lox^ mice infected with L. major developed similar kinetics of lesion development and resolution as those observed in C57BL/6 mice genetically resistant to two strains of L. major. In contrast, control IL-4Rα^−/lox^ (WT) and IL-4Rα^−/−^ BALB/c mice developed progressive lesion swelling leading to necrosis during the acute and chronic phases of disease as expected. Lck^cre^IL-4Rα^−/lox^ BALB/c and C57BL/6 mice also resisted secondary parasite challenge, unlike WT mice, which showed no signs of footpad pathology. A similar resistant phenotype to L. major infection was also noted in an independent line of mice in which IL-4Rα is efficiently deleted from CD4, CD8, NK–T, and γδ T cells (unpublished data), indicating that IL-4–responsive CD4^+^ T cells control susceptibility to L. major infection, and that the resistant phenotype is not associated with Cre activity in T cells or hypothetical mutations introduced by the transgene. Together, our study demonstrates that clinical immunity can be achieved in mice on a susceptible BALB/c background by abrogating IL-4Rα responsiveness on CD4^+^ T cells while retaining IL-4/IL-13–mediated function on non-CD4^+^ T cells.

IL-10 is a potent suppressor of macrophage activation [37], can abolish IFN-γ/LPS–induced killing of L. major by macrophages [38,39], and can suppress development of DTH responses [40]. In agreement, L. major–infected C57BL/6 and Lck^cre^IL-4Rα^−/lox^ mice developed DTH responses to SLA, inhibited by coadministration of IL-10. In contrast, DTH responses in WT mice were absent. Neutralization of IL-10 signaling allowed WT mice to mount a significant response to SLA. Together, DTH data demonstrated that IL-10 produced in response to SLA in susceptible mice was able to suppress protective cell-mediated immune responses.

IL-10 production is increased in BALB/c mice compared with resistant mice [41], can regulate parasite survival in resistant C57BL/6 mice [1,42], and is a susceptibility factor for L. major infection [31,39]. In agreement, the draining LNs of infected resistant Lck^cre^IL-4Rα^−/lox^ and C57BL/6 mice contained reduced numbers of CD4^+^ IL-10–secreting cells (4- and 9-fold less, respectively) compared with WT mice. Variable amounts of IL-10 staining were observed in the non-CD4^+^ T cell population; however, this was found to be nonspecific (Figure 4A). Increased IL-10 secretion was also observed in anti-CD3–stimulated CD4^+^ T cells derived from WT mice compared with T cells derived from Lck^cre^IL-4Rα^−/lox^ and C57BL/6 mice (not shown). IL-10 production by macrophages [43] and CD4^+^ T cells [31] has been linked to susceptibility to L. major infection. Using our assay system, IL-10–secreting cells were identified as CD4^+^ T cells. IL-10–producing CD4^+^ T cells have been implicated in controlling L. major parasite survival/infection in genetically resistant C57BL/6 mice. CD4^+^CD25^+^FoxP3^+^ IL-10–producing natural T regulatory cells (Tregs) have been elegantly shown to control parasite survival [44,45]. More recently, a novel disease controlling FoxP3^−^ IL-10/IFN-γ–coproducing Th1 cell population has been identified [46]. The role for Tregs in control of L. major is unclear in BALB/c mice and potentially obscured by the predominant polarized Th2 response. The moderately specific method of Treg depletion using anti-CD25 antibody has produced contradictory results either enhancing [47] or reducing [48] susceptibility to L. major infection. Certainly, IL-4 has the ability to enhance the proliferation and function of CD4^+^CD25^+^ T cells in BALB/c mice [49,50]. However, the generation of CD4^+^FoxP3^+^ T cells was unaffected by IL-4Rα deficiency (unpublished data). Therefore, while not excluding a role for macrophage IL-10 production [43], our data suggest that IL-10 is predominantly produced by activated/effector T cells or Tregs, and further characterization of the CD4^+^IL-10^+^ T cells is ongoing.

The absence of IL-4Rα specifically on CD4^+^ T cells resulted in consistently higher levels of IFN-γ secretion by CD4^+^ T cells compared with WT mice. However, as previously shown, induction of increased IFN-γ responses alone does not guarantee control of L. major infection. Substantially increased L. major–specific CD4^+^ T cell IFN-γ production was observed in macrophage/neutrophil-specific IL-4Rα–deficient mice when compared with WT controls. However, infection also induced a potent polarized Th2 response, and lesion development was delayed but uncontrolled [9]. In contrast, in the absence of a polarized Th2 response, increased IFN-γ production correlated with protection against infection in Lck^cre^IL-4Rα^−/lox^ and C57BL/6 mice. Significant DTH responses upon injection of SLA into the footpad were observed as early as 3 wk after infection in Lck^cre^IL-4Rα^−/lox^ and C57BL/6 mice, but not in WT mice (unpublished data). Sustained tuberculin-like DTH responses are driven by IL-12–induced IFN-γ–producing Th1 cells [34,51], resulting in macrophage recruitment and activation, and are indicative of protective cell-mediated immune responses against intracellular pathogens. This was confirmed by increased IL-12 protein detected in tissue lysate of footpads of resistant mice compared with WT mice 24 h after DTH induction. Furthermore, increased levels of IFN-γ secretion were associated with increased expression of iNOS mRNA/parasite in infected footpads. Together, these results demonstrate that in the absence of IL-4Rα signaling on CD4 T cells, a polarized Th2 response, and IL-10 production, protective Th1 immune responses during cutaneous leishmaniasis result in effective macrophage activation and intracellular parasite elimination.

IL-4Rα^−/−^ mice are susceptible to L. major infection in the acute [31] or the chronic [20] phase. Despite the absence of Th1 downregulatory signals through the IL-4Rα, IL-4Rα^−/−^ mice do not produce increased amounts of IFN-γ following L. major infection when compared with WT controls [7]. Resistance to L. major in Lck^cre^IL-4Rα^−/lox^ mice has therefore revealed the protective role of IL-4/IL-13–responsive non-CD4^+^ T cells in control of infection in BALB/c mice. Crucial to resistance in Lck^cre^IL-4Rα^−/lox^ mice is CD4^+^ T cell IL-4Rα–independent IL-4 production. Not only induced following L. major infection [7,31] in IL-4Rα^−/−^ mice, IL-4Rα–independent IL-4 production has been observed in response to Nippostrongylus brasiliensis [52] and Schistosoma mansoni [53] infections and following immunization with protein precipitated in alum [54]. As our study suggests, IL-4Rα–independent IL-4 production in Lck^cre^IL-4Rα^−/lox^ mice drives the induction of protective responses by non-CD4^+^ T cells. Both IL-4 and IL-13 are able to indirectly promote protective Th1 responses. Elegant experiments have demonstrated that IL-4 is able to instruct DCs to produce IL-12 and subsequent protection from L. major infection in BALB/c mice [55]. Furthermore, IL-4 is required for protective type 1 responses to *Candida* [56]. IL-13 can prime monocytes for IL-12 production [57] and drive protective cell-mediated immune responses during listeriosis [58]. Indeed, levels of IL-12p35 mRNA were increased in draining LNs of Lck^cre^IL-4Rα^−/lox^ and C57BL/6 mice by 3 wk after infection (Figure 5A), coincident with increased DTH responses (unpublished data). As macrophage IL-12 production is actively downregulated by L. major [18], it is likely that increased IL-12p35 mRNA levels in the LNs at 3 wk after infection were produced by DCs. In agreement, infected DCs appear in draining LNs in two waves; the first transient wave peaks at 24 h, and the second commences 15–21 d after L. major infection [59]. Therefore, IL-4Rα–independent IL-4 production and subsequent IL-12 production by DCs in the absence of Th2 polarization may explain the protection of Lck^cre^IL-4Rα^−/lox^ from L. major infection. Furthermore, the protective effect of IL-4 signaling in non-CD4^+^ T cells may also explain the requirement for IL-4 in effective treatments against visceral leishmaniasis [60,61].

In summary, in the absence of a polarized Th2 response where non-CD4^+^ T cells retain IL-4/IL-13 responsiveness, increased protective immune responses are induced by 3 wk in Lck^cre^IL-4Rα^−/lox^ mice. As IL-12 may also negate Treg cell action on activated T cells [62], this regulation is likely to enhance beneficial Th1 responses and immunity following L. major infection in Lck^cre^IL-4Rα^−/lox^ mice, possibly reflecting a similar scenario in the healer C57Bl/6. In contrast, IL-4Rα expression on CD4^+^ T cells allows Th2 polarization and induction of IL-10 production in the nonhealer BALB/c strain. As a consequence, Th1 responses and protective macrophage effector functions are downregulated, IL-10 is upregulated, and subsequently, BALB/c mice succumb to L. major infection in the acute phase. In conclusion, where CD4^+^ T cells are unable to respond to IL-4, IL-4/IL-13 signaling in non-CD4^+^ T cells is beneficial in BALB/c mice following infection with *L. major.*

## Materials and Methods

### Generation and genotyping of Lck^cre^IL-4Rα^−/lox^ BALB/c mice.

Transgenic Lck^cre^ mice [28] back-crossed to BALB/c for nine generations were intercrossed with IL-4Rα^−/−^ and IL-4Rα^lox/lox^ mice to generate Lck^cre^IL-4Rα^−/lox^ BALB/c mice. WT littermates were used as controls in all experiments. Mice were genotyped as described previously [24]. All mice were housed in specific pathogen–free barrier conditions at the University of Cape Town, South Africa, and used in accordance with University ethical committee guidelines. All experimental mice were age and sex matched and used between 8–12 wk of age.

### Analysis of IL-4Rα deletion efficiency.

DNA was prepared from CD3^+^CD4^+^ and CD19^+^ sorted LN cells from Lck^cre^IL-4Rα^−/lox^, WT, or IL-4Rα^−/−^ mice using a FACsvantage flow cytometer (BD, http://www.bd.com) to >99% purity. A standard curve was prepared from serial 10-fold DNA dilutions of cloned IL-4Rα exon 5 and exon 8 DNA. Primers: exon 5 forward 5′ AACCTGGGAAGTTGTG 3′, exon 5 reverse 5′ CACAGTTCCATCTGGTAT 3′; exon 8 forward 5′ GTACAGCGCACATTGTTTTT 3′, exon 8 reverse 5′ CTCGGCGCACTGACCCATCT 3′.

### Detection of parasite DNA.

DNA was prepared from homogenized tissues samples. A DNA standard curve was prepared from serial 10-fold parasite DNA dilutions in PBS. L. major kinetoplast primers used: forward 5′ CGCCTCCGAGCCCAAAAATG 3′ and reverse 5′ GATTATGGGTGGGCGTTCTG 3′. Real-time PCR amplification and data analysis performed using the “Fit Points” and “Standard Curve” methods as described previously [63].

### Flow cytometry.

IL-4Rα was detected by anti-IL-4Rα–PE (M-1; BD), and leukocyte subpopulations were identified using anti-CD19 (1D3), anti–δ-TCR (GL3), anti-CD11^c^ (HL3), anti-F4/80, anti–I-A^d^ (AMS-32.1), anti-CD11^b^ (M1/70) (all from BD), anti-CD3 (145–2C11), anti-CD4 (GK1.5), and anti-CD8 (53.6.72) mAbs, which were purified from hybridoma supernatants by protein G sepharose (Amersham Biosciences, http://www.amersham.com) and labeled with FITC or biotin. Biotin-labeled antibodies were detected by streptavidin–allophycocyanin (BD). Dead cells were stained by 7-AAD and excluded from analysis (Sigma, http://www.sigmaaldrich.com). Acquisition was performed using FACSCalibur, and data were analyzed by Cellquest (BD).

### T cell proliferation.

CD4^+^ T cells, positively selected by anti-CD4 Dynabeads (Invitrogen, http://www.invitrogen.com) to a purity of >85% as described [7], were stimulated with serial dilutions of IL-4, IL-13, or IL-2 (BD) in complete IMDM containing 10% FCS, penicillin, and streptomycin, 1 mM sodium pyruvate, NEAA (Invitrogen), 10 mM HEPES, and 50 μM β2-ME (Sigma). After 48 h of incubation at 37 °C and 5% CO_2_, cells were pulsed with 1 μCi (0.037 MBq) [^3^H] thymidine (Amersham Biosciences) for a further 18 h. [^3^H] incorporation was measured in a liquid scintillation counter.

### In vitro Th2 differentiation.

In vitro Th1/Th2 differentiation of purified CD4^+^ T cells was induced as described previously [7].

### Suppression of macrophage-derived NO secretion.

Suppression assay was performed as described [20]. Briefly, adherent macrophages derived from peritoneal exudate cells elicited with 3% Brewers thioglycollate (Difco Laboratories, http://www.bd.com/ds) were incubated for 16 h with medium or with IL-4, IL-13, or IL-10 at 1,000 U/ml (R&D Systems, http://www.rndsystems.com). Cells were subsequently stimulated with LPS (15 ng/ml; Sigma) and IFN-γ (100 U/ml; BD) and NO was measured by Griess reaction after 48 h.

### Induction of IgE response.

Mice were immunized subcutaneously with 10 μg of OVA in CFA (Sigma) and boosted at 7 and 14 d with OVA intraperitoneally. IgE production was detected as described previously [20].

### L. major infection.

L. major LV39 (MRHO/SV/59/P) and MHOM/IL/81/FEBNI strains were maintained by continuous passage in BALB/c mice and cultured in vitro as described previously [20]. Mice were inoculated subcutaneously with 2 × 10^6^ stationary phase metacyclic promastigotes into the left hind footpad in a volume of 50 μl HBSS (Invitrogen). Swelling was monitored every week up to a maximum of 40 wk using a Mitutoyo pocket thickness gauge (http://www.mitutoyo.com). For reinfection studies, 6 wk after primary infection, mice were injected subcutaneously with 2 × 10^6^ stationary phase metacyclic promastigotes into the contralateral footpad. Footpad swelling was monitored for 18 wk.

### Detection of viable parasite burden.

Infected organ cell suspensions were cultured in Schneider's culture medium (Sigma). Parasite burden was estimated according to a previously described limiting dilution method [20].

### Quantification of iNOS and IL-12p35 RNA.

Total RNA from footpad or LN was purified using mini-elute columns (Qiagen, http://www.qiagen.com) and cDNA was generated using the Inprom-II re-verse transcription system (Promega, http://www.promega.com). Primers pairs used to detect IL-12p35 message: forward 5′-GATGACATGGTGAAGACGGCC-3′, and reverse 5′-GGAGGTTTCTGGCGCAGAGT-3′. iNOS message forward 5′-AGCTCCTCCCAGGACCACAC-3′, and reverse 5′-ACGCTGAGTAC CTCATTGGC-3′. Data analysis was performed using the “Fit Points” and “Standard Curve” methods using *beta-2-microglobulin* as a housekeeping gene.

### DTH reaction.

Mice were inoculated subcutaneously with 10 μg SLA into the right hind footpad alone or with 0.5 μg mouse rIL-10 or 1.5 μg anti–IL-10Rα (R&D Systems). Footpad swelling was measured every 24 h. Footpads were homogenized, and lysates were taken 24 h after induction of DTH.

### Antigen-specific restimulation.

CD4^+^ T cells were positively selected using anti-CD4 Macs beads (Miltenyi Biotec, http://www.miltenyibiotec.com) to a purity of >90% according to the manufacturer's instructions. Thy1.2-labeled splenocytes were T cell depleted by complement-mediated lysis (Cedarlane, http://www.cedarlanelabs.com) to produce antigen-presenting cells (APCs). APCs fixed with mitomycin C (50 μg/ml, 20 min at 37 °C) and washed extensively in complete IMDM. A total of 2 × 10^5^ purified CD4^+^ T cells and 1 × 10^5^ APCs were cultured with SLA at 50 μg/ml, supernatants were collected after 48 h, and cytokines were analyzed as previously described [20].

### Cytokine detection in tissue homogenates.

IFN-γ and IL-4 were detected in footpad tissues using the method previously described [24].

### Intracellular staining.

L. major–infected mice; popliteal LN cells at 2 × 10^5^ cells/well were stimulated with SLA (5 μg/ml) for 24 h. Cultures were supplemented with monensin (2 μM) for the final 4 h of culture. Cells were stained with anti-CD4 FITC (mAb, GK1.5), fixed, permeabilized, and stained with anti–IL-10 APCs (BD).

### Statistics.

Values are given as mean ± SD and significant differences were determined using Student's *t* test (Prism software, http://www.prism-software.com).

## Supporting Information

Figure S1 Variable Deletion Efficiency of IL-4Rα on CD8^+^ T Cells WT (black line), IL-4Rα^−/−^ (gray line), and Lck^cre^IL-4Rα^−/lox^ BALB/c mice (dashed line) peripheral blood lymphocytes were stained for expression of IL-4Rα. CD8^+^ T cells were identified using anti-CD3 and anti-CD8.
(30 KB PDF)

## Abbreviations

APC: antigen-presenting cell
DC: dendritic cell
DTH: delayed type hypersensitivity
FACS: fluorescence-activated cell sorter
IFN: interferon
iNOS: inducible nitric oxide synthase
LN: lymph node
NO: nitric oxide
SLA: soluble *L. major* antigen
Th: T helper
Treg: T regulatory cell
WT: IL-4Rα^−^^/lox^
