# Supplementary material for: Deletion of IL-4Rα on CD4 T Cells Renders BALB/c Mice Resistant to Leishmania major Infection
Source: PLoS Pathog. 2007 May 11;3(5):e68. doi: 10.1371/journal.ppat.0030068 (PMC1867380; doi:10.1371/journal.ppat.0030068)
Supplement: Supplementary file 1 [file ppat.0030068.sg001.pdf]

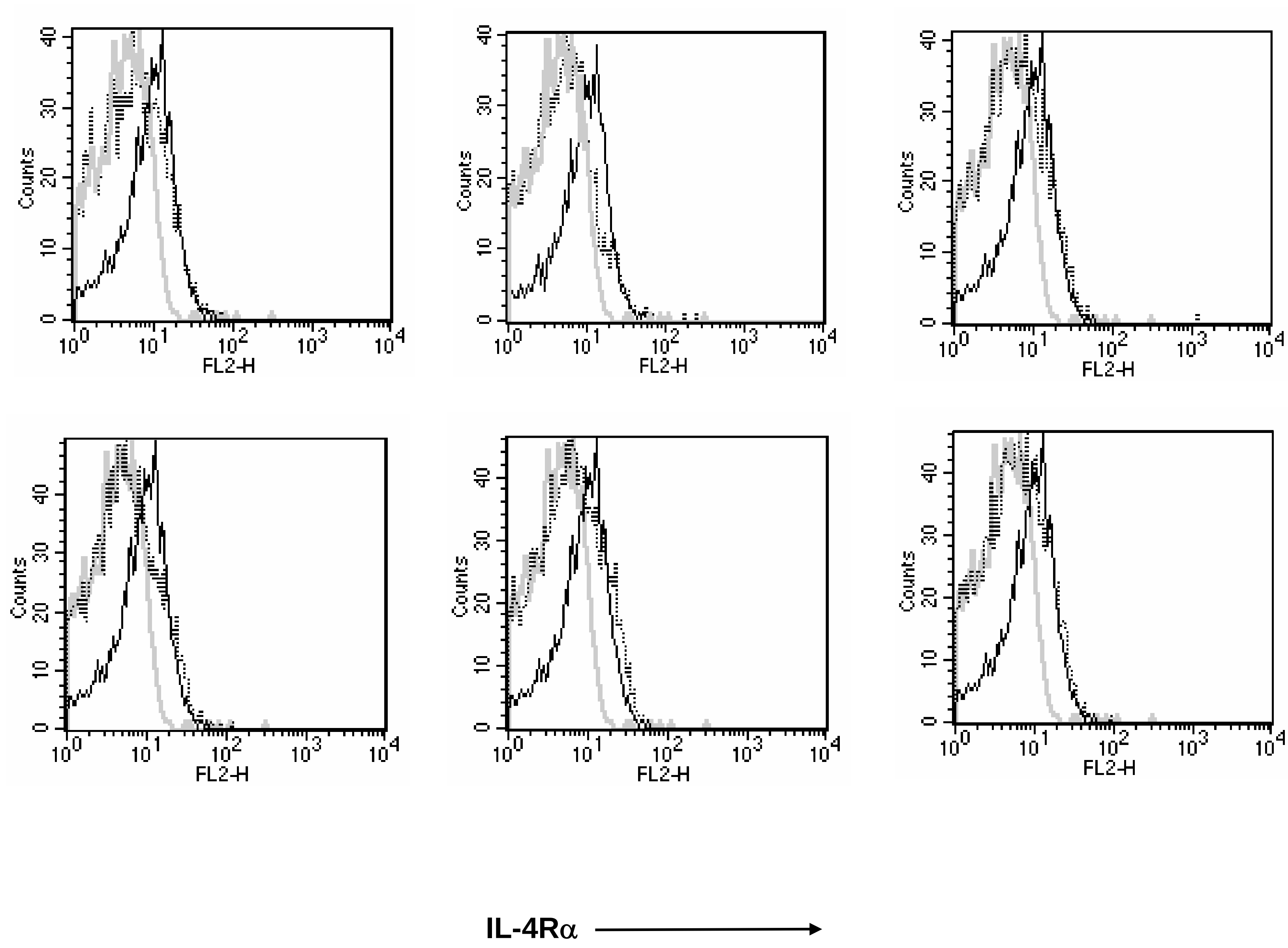

Counts
FL2-H
IL-4Rα
